# Low-Dose Radiation Therapy for COVID-19: Promises and Pitfalls

**DOI:** 10.1093/jncics/pkaa103

**Published:** 2020-11-19

**Authors:** Bhanu P Venkatesulu, Scott Lester, Cheng-En Hsieh, Vivek Verma, Elad Sharon, Mansoor Ahmed, Sunil Krishnan

**Affiliations:** 1 Department of Radiation Oncology, Loyola University Stritch School of Medicine, Chicago, IL, USA; 2 Department of Radiation Oncology, Mayo Clinic, Rochester, MN, USA; 3 Department of Immunology, MD Anderson Cancer Center, Houston, TX, USA; 4 Department of Radiation Oncology, MD Anderson Cancer Center, Houston, TX, USA; 5 Radiation Research Program, Division Cancer Treatment and Diagnosis, National Cancer Institute, Bethesda, MD, USA; 6 Cancer Therapy Evaluation Program, Division Cancer Treatment and Diagnosis National Cancer Institute, Bethesda, MD, USA; 7 Department of Radiation Oncology, Mayo Clinic Florida, Jacksonville, FL, USA

## Abstract

The coronavirus disease-2019 (COVID-19) pandemic caused by SARS-CoV-2 has exacted an enormous toll on healthcare systems worldwide. The cytokine storm that follows pulmonary infection is causally linked to respiratory compromise and mortality in the majority of patients. The sparsity of viable treatment options for this viral infection and the sequelae of pulmonary complications have fueled the quest for new therapeutic considerations. One such option, the long-forgotten idea of using low-dose radiation therapy, has recently found renewed interest in many academic centers. We outline the scientific and logistical rationale for consideration of this option and the mechanistic underpinnings of any potential therapeutic value, particularly as viewed from an immunological perspective. We also discuss the preliminary and/or published results of prospective trials examining low-dose radiation therapy for COVID-19.

Coronaviruses belong to a family of RNA viruses that cause diseases in animals and less commonly in humans. However, 7 coronaviruses are known to infect humans: 2 alpha coronaviruses, 229E and NL63; and 5 beta coronaviruses, OC43, HKU1, Middle East Respiratory Syndrome, Severe Acute Respiratory Syndrome (SARS-CoV), and SARS-coronavirus-2 (SARS-CoV-2). The human coronaviruses were initially identified in the early 1960s coinciding with the discovery of the first antiviral drug, idoxuridine. Since then, the SARS and Middle East Respiratory Syndrome outbreaks of 2002-2003 and 2012, respectively, have brought these viruses back into focus owing to the estimated case fatality rates of 10% and 30%, respectively. December 2019 heralded the dawn of a novel, rapidly spreading virus from the coronavirus family, SARS-CoV-2 (1), which has (at the time of writing) infected 21 million people around the world, leading to nearly 750 000 deaths from coronavirus disease-2019 (COVID-19) (2,3). The case fatality rate has ranged from 1% to 10% in different healthcare settings for the general public infected with SARS-CoV-2, 10% to 20% in hospitalized oxygen-dependent patients, and even higher in intubated patients (4-7).

Despite the many years since the original outbreaks of these viruses, the only drugs that have shown a modest clinical benefit are remdesivir and dexamethasone. In the Adaptive COVID-19 Treatment Trial study, a double-blind randomized placebo-controlled trial of remdesivir in hospitalized COVID-19 patients with lower respiratory infection, a 10-day course of remdesivir resulted in shorter recovery time (median = 11 vs 15 days) and no improvement in overall survival albeit a trend towards a lower 14-day mortality rate (7.1% vs 11.9%) than placebo (8). A preprint report of the RECOVERY trial, which randomly assigned 6425 hospitalized COVID-19 patients 2:1 to standard of care vs 6 mg dexamethasone daily for up to 10 days, noted that steroid treatment statistically significantly reduced 28-day mortality in patients undergoing invasive mechanical ventilation (29.0% vs 40.7%) and those requiring oxygen (21.5% vs 25%) (9). Multiple treatment strategies have been evaluated for COVID-19, including chloroquine and hydroxychloroquine (membrane fusion and endocytosis inhibitors), arbidol (viral envelope membrane fusion inhibitor), remdesivir, ribavirin, and favipiravir (viral RNA-dependent RNA polymerase inhibitors), lopinavir and darunavir (chymotrypsin-like protease inhibitors), and tocilizumab and sarilumab (interleukin-6 [IL-6] inhibitors) (10). None of these pharmacological interventions has statistically significantly reduced the intensive care admission rates or mortality rates or improved any measurable outcomes. As a result, there is a pressing need for newer and/or alternative treatment strategies.

One consideration is to revisit the long-forgotten idea of using low-dose radiation therapy (LDRT) for patients with lobar and interstitial pneumonia. Historically, from 1905 to the mid-1940s, LDRT was used in the treatment of nonresolving pneumonias, and anecdotal evidence suggests that this may have improved survival and provided rapid palliation of respiratory symptoms (11). LDRT was rightly supplanted by the development of antibiotic and supportive medications. However, the lack of effective pharmacotherapy for this SARS-CoV-2 has prompted a reassessment of this old paradigm. Although the mechanism of action is yet to be defined, evidence from multiple contemporary preclinical and clinical experiences with LDRT could be used to generate testable hypotheses in the current COVID-19 crisis. In this review, we outline the rationale for considering the use of LDRT in moderately symptomatic patients with SARS-CoV-2 infection.

## Clinical Features and Biology of COVID-19

SARS-CoV-2 is transmitted through aerosol spread, whereby the virus gains entry into cells through the angiotensin-converting enzyme carboxypeptidase and the transmembrane protease serine cell surface receptors; these are highly expressed in the nasal mucosa, type 2 pneumocytes, and enteral goblet cells (12). Viral entry coupled with rapid replication within cells results in pattern recognition receptors (PRRs) sensing viral nucleic acid motifs. In addition to nucleic acid motifs, other hallmarks of the pathogen-associated molecular patterns sensed by PRRs are structural proteins like the nucleocapsid, spike glycoproteins, membrane and envelope proteins, and nonstructural proteins because they are broadly shared by different viruses and contribute to infectivity. Sensing of pathogen-associated molecular patterns by PRRs culminates in transcription of a variety of proinflammatory interferon regulatory factors and nuclear factor kappa B (NF-κB). This leads to the upregulation of type I and III interferons and interferon stimulated genes as well as transmigration and homing of leukocytes mediated by chemokine secretion (13,14). Successful containment of the infection results in downregulation of this inflammatory response and resumption of normal homeostatic functioning of all cells in the tissue microenvironment. However, unchecked inflammation and unabated release of cytokines and chemokines results in a cytokine release syndrome that is the proximate pathophysiological cause of rapid clinical deterioration seen in SARS-CoV-2 infection. Not only does this result in normal tissue injury, but the ineffectiveness of the host antiviral response in eradicating the infection may also exert selection pressure on the viral machinery and drive the evolution of viral escape mechanisms.

Consistent with this notion of unrestrained inflammation, the respiratory complications arising from COVID-19 have been mechanistically correlated with macrophage polarization to the proinflammatory M1 phenotype (15). Autopsy studies of patients with severe SARS-CoV-2 infection as well as necropsy of experimentally infected nonhuman primates have documented immune-mediated injury to alveolar epithelial cells, hyperplasia of type II pneumocytes with hyaline membrane formation accompanied by fibroblastic consolidation, and diffuse alveolar damage (16,17).

A cardinal hematological feature that is also a prognostic factor for COVID-19 is lymphopenia. Notably, elevated IL-6 and serum C-reactive protein (CRP) (triggered by IL-6) are markers for clinical deterioration and ventilatory support in COVID-19. Elevated levels of IL-6 lead to downstream signaling via Janus kinase and signal transducer and activator of transcription 3 activation, which in turn leads to activation of neutrophils, macrophages, and natural killer cells (18,19). In a study of 522 patients from China assessing the immune profile of patients with COVID-19, researchers reported that in patients with severe disease, the total T-cell count (including both CD4+ and CD8+ subsets) was severely reduced. Importantly, this also correlated with poorer survival outcomes. There was also a reciprocal negative correlation between T-cell count and the serum concentration of cytokines such as IL-6, IL-10, and tumor necrosis factor (TNF)-α. These findings suggest that severe T-cell exhaustion and widespread cytokine activation may be prime causes of mortality in patients with severe COVID-19 disease (20).

From a clinical standpoint, patients with SARS-CoV-2 infection can present with diverse manifestations ranging from asymptomatic cases to mild symptomatology with fever, cough, and myalgia to more overt symptomatology with pneumonia, sepsis, acute respiratory distress syndrome, and respiratory failure. Patients with mild disease usually have minimal symptoms, such as fever, cough, myalgia, and diarrhea with spontaneous resolution of symptoms. However, patients with moderate or severe disease are usually hospitalized and require close monitoring. Individuals with severe disease have increasing oxygen requirements (oxygen saturation <94% on room air) with elevated IL-6, CRP, d-dimer, and ferritin; imaging findings often reveal infiltrates scattered throughout more than 50% of the lung. Older patients and those with comorbidities such as diabetes mellitus, hypertension, obesity, cardiovascular disease, and cancer are at highest risk of requiring ventilator support and dying secondary to acute respiratory distress syndrome (21). Some evidence suggests that a dysfunctional and compromised immune response in patients with preexisting comorbidities might explain the greater mortality and morbidity seen in these patients (18).

## Radiation Therapy and the Immune Response

Radiation is a double-edged sword with regard to the immune system; LDRT and high-dose RT have differential effects on the immune subsets. In the preclinical context, LDRT exerts effects on endothelial cells, leukocytes, macrophages, and dendritic cells. Collectively, these serve to create an antiinflammatory milieu, as mechanistically described below.

Endothelial cells play an important role in regulating inflammation through expression of cell surface adhesion molecules (eg, E-selectin) that contribute to leukocytic homing to inflamed vessels. When Rodel et al. (22) assessed the effect of LDRT on the interaction between human or murine endothelial cells and peripheral blood mononuclear cells (PBMCs), LDRT doses as low as 0.3-0.6 Gy impaired the adhesion and migration kinetics of PBMCs by lowering the expression of E-selectin. This effect correlated with increased expression of the anti-inflammatory cytokine transforming growth factor beta 1 (TGF-β1) (22,23). Rodel et al. (22) also reported that LDRT between 0.5 Gy and 1 Gy caused a statistically significant downregulation in CCL20 release (which normally plays an important role in the transmigration of B cells through the inflamed endothelium), which in turn is modulated by TGF-β1. This downregulation in CCL20 therefore correlated with reduction in leukocytic adhesion to endothelial cells, because downregulation of CCL20 prevents leukocyte chemotaxis to the site of injury (24).

LDRT is also known to induce apoptosis of PBMCs, resulting in reduced TNF-α and IL-1 production, reduced L-selectin expression, and increased anti-inflammatory cytokine IL-10 production. Arenas et al. (25) treated mice with LDRT (0.1, 0.3, and 0.6 Gy) and reported upregulation of TGF-β expression with corresponding downregulation of leukocyte recruitment. Kern et al. (26,27) reported that doses of 0.3-0.7 Gy LDRT increased apoptosis of PBMCs with reduced expression of L-selectin, mitogen activated protein (MAP) kinases, and protein kinase B, all of which are involved in PBMC proliferation. Tsukimoto et al. (28) reported that 0.5-1 Gy gamma irradiation caused RAW264.7 macrophage cells to reduce the secretion of TNF-α in response to lipopolysaccharide as well as reduce the activity of p38 MAP kinase (which predominantly increases proinflammatory cytokine secretion).

**Figure 1.**
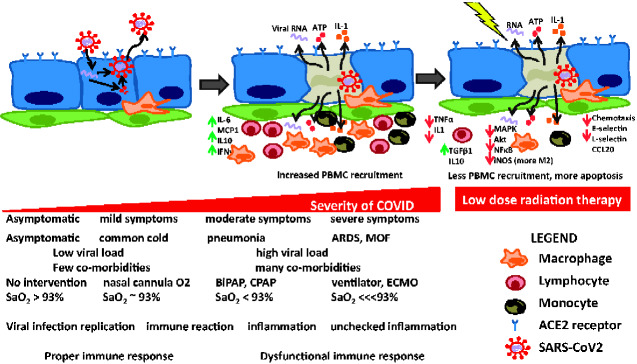
Schematic representation of viral infection, replication, and immune effects of Severe Acute Respiratory Syndrome-coronavirus-2 (SARS-CoV-2) infection and the possible mechanisms of low-dose radiation therapy. Depicted on the **top** are cartoons of lung epithelial cells in **blue**, endothelial cells in **green**, and resident macrophages in **orange**. The cartoon on the **left** illustrates early infection by the virus (**red circle with spikes**), internalization, replication, reformation, and release. In the **middle** is a depiction of release of pathogen-associated molecular pattern (PAMP) hallmarks, including ATP, viral RNA, and interleukin-1 (among others); destruction of the infected cell (now **gray**); and recruitment of peripheral blood mononuclear cells (PBMCs), adherence to endothelial cells during this chemotaxis, and elaboration of host of proinflammatory cytokines (**green arrows**). On the **right** is a depiction of potential consequences of low-dose radiation of this immune-dysregulated lung microenvironment where there is less PBMC recruitment, less chemotaxis, less endothelial adherence because of downregulation of E/L-selectins, more apoptosis and less proliferation of recruited PBMCs, and a shift in the proinflammatory cytokine milieu towards more of an anti-inflammatory one. Also depicted on the **bottom** panel are features of COVID as the severity of disease increases (from left to right): clinical manifestations, interventional approaches, and viral and immune responses. ACE2 = angiotensin converting enzyme 2; ARDS = acute respiratory distress syndrome; BiPAP = bilevel positive airway pressure; CPAP = continuous positive airway pressure; ECMO = extracorporeal membrane oxygenation; MOF = multi-organ failure; SaO_2_ = oxygen saturation.

LDRT also exerts notable effects on macrophages. Lodermann et al. (29) reported the reduced secretion of IL-1β with doses of 0.5-0.7 Gy in human THP-1–derived macrophages; this, in turn, translated to a reduction in activity of NF-κB, which led to attenuated expression of both the p38 MAP kinaseand AKT pathways. Schuae et al. (30) showed that LDRT (0.3-0.6 Gy) reduced oxidative burst activity in RAW 264.7 macrophages, resulting in diminished production and release of free radicals. Hildebrandt et al. (31) assessed the impact of varying radiation doses (0, 0.3, 0.6, 1.25, 2.5, 5, and 10 Gy) on resting as well as activated macrophages. They found that with radiation doses up to 1.25 Gy, inducible nitric oxide synthase (iNOS) was downregulated (resulting in reduction of nitric oxide production), whereas upregulation of iNOS was seen with doses more than 1.25 Gy. These reports suggest that lower doses of LDRT drive an anti-inflammatory M2 phenotype by suppressing iNOS, whereas higher LDRT doses lead to a proinflammatory-activated M1 macrophage phenotype with iNOS pathway activation (31) (Figure 1).

These results are consistent with the well-recognized phenomenon of hyper-radiation sensitivity (HRS) where a biphasic response is seen with LDRT. Mammalian cell lines have shown that single doses of LDRT of less than 10 cGy result in heightened sensitivity to radiation and that radiation doses beyond 0.5 Gy result in relative radio-resistance (32). In normal cells such as skin and peripheral blood lymphocytes, 0.5 Gy causes ATM autophosphorylation resulting in activation of DNA repair programs; however, in cancer cells, the dose to activate ATM pathways is greater than 1 Gy. The evolving evidence suggests that HRS is due to a lack of activation of ATM autophosphorylation pro-survival pathways. Based on this preclinical evidence, HRS has been used in the clinic to prime cells for greater efficacy and less toxicity when combined with chemotherapy. Clinical trials have been completed in a number of disease sites, including head and neck, recurrent ovarian, pancreatic and small bowel, endometrial, breast, recurrent or progressive brain, and recurrent lung cancers (33), and as an experimental arm with dual checkpoints in an ongoing study in metastatic non-small cell lung cancer (NCT02888743).

On the other hand, some evidence suggests that LDRT may prime and activate macrophages. Klug et al. showed in murine cancer models that single radiation doses of 0.5-0.6 Gy result in normalization of aberrant vasculature with recruitment of tumor-specific T cells as well as macrophages. These macrophages were predominantly of the proinflammatory M1 phenotype expressing iNOS, the promoter of which contains the NF-κB consensus sequence (34). In principle, this might result in more efficient antiviral responses being mounted, but because the proximate pathophysiological cause of mortality and morbidity in severe COVID-19 patients is the cytokine surge characterized by influx of classically activated M1 macrophages, this may be counterproductive.

A similar dichotomy might exist with the effect of LDRT on lymphocytes. Because clinical COVID-19 data suggest that T-cell exhaustion and reduction in numbers are more common in patients with severe SARS-CoV-2 infection, any further depletion by LDRT may be detrimental. RT for cancer treatment frequently causes low-dose radiation exposure of secondary lymphoid organs; the resulting lymphopenia can have a detrimental effect on tumor control and patient survival (35). Although these doses are still larger than the LDRT doses outlined above, any depletion of an already low lymphocyte count during severe COVID-19 disease may be worrisome. Again, LDRT in the mouse model of pancreatic islet cell carcinoma by Klug et al. (34) suggests exactly the opposite, wherein LDRT increased tumor infiltration by CD8+ T cells. It thus remains unclear what the dominant effect of LDRT on T cells might be. Consistent with this mechanistic uncertainty is a preclinical report from the 1940s suggesting that mortality from experimental swine influenza viral pneumonias in mice was statistically significantly reduced (by one-third) when they were treated with x-ray therapy (100 r) 48 hours before inoculation of the virus but not when treatment was initiated 24 hours after inoculation (36). The explanation offered was that established viral pneumonias are more refractory to treatment and that there is a lag of approximately 48 hours for radiation to exert its maximal effect via activation of reticuloendothelial cells for better phagocytosis.

Clearly, a nuanced understanding of lung tissue and immune biology, radiation dose-volume considerations that drive clinicopathological changes, mechanisms of injury, and biomarkers that predict the course of disease is direly needed (37). Given sufficient time, the scientific rationale and mechanistic underpinnings of LDRT undoubtedly need to be fortified by systematic preclinical studies before advancing to well-designed randomized clinical trials with correlative endpoints and predictive biomarkers. This desire for sound preclinical scientific evidence must be balanced against the difficulty of generating mouse models of SARS-CoV-2 pneumonia in a BSL3 laboratory, the practical and ethical challenges of colocating radiation facilities and these BSL3 laboratories, and the unproven translatability of these models to human disease.

## Clinical Precedent for LDRT Use in Pneumonias

More than 700 patients have been reported in historical medical publications as having received LDRT for pneumonias that failed to abate after other therapies. In the largest series of LDRT for pneumonia, Powell et al. (38) noted that x-ray therapy for lobar pneumonia and bronchopneumonia reduced the mortality rate to 5% and 13%, respectively, compared with 30% seen in the 1930s. The response to therapy in most cases was rapid, with resolution of symptoms within hours (38,39). Oppenheimer (11,40) reported clinical improvement of interstitial pneumonias in 33 of 36 pediatric patients as well as 45 of 56 adult patients treated with 30-90 Roentgen (approximately 0.3-0.7 Gy), with earlier treatment resulting in better outcomes than when treatment was delayed by more than 2 weeks. Additionally, leukocyte counts did not decrease following LDRT for pneumonia even in the setting of preexisting leukopenia. Notably, the pulmonary consolidation disappeared within 3-5 days after RT. However, these studies were observational single-arm studies without a control group, hence burdened by unavoidable limitations because of their retrospective nature. Moreover, the studies predate the antiviral and antibiotic era, the x-ray therapy used was primitive compared with contemporary RT, and these studies did not delineate the correlates of response to therapy. As a result, not surprisingly, the advent of sulfonamide antibiotics in the early 1940s eroded interest in x-ray therapy for pneumonias.

## Rationalizing the Use of LDRT for COVID-19 Treatment

The foregoing accounts of preclinical and clinical evidence and rationale for considering LDRT for COVID-19 provide a glimpse of the promises and pitfalls associated with advocating for LDRT during the current health crisis.

LDRT-related toxicities are expected to be mild, because there is a long-standing precedent for the use of whole lung radiation of 5-8 fractions of 1.5 Gy per fraction in Ewing’s sarcoma and Wilms tumor along with whole-body radiation for bone marrow ablation in transplant-conditioning regimens. These are generally considered safe and have an acceptable safety profile (41,42). Moreover, doses as low as 0.3-0.6 Gy are unlikely to cause any appreciable early side effects such as pneumonitis, esophagitis, myelitis, cardiac injury, or late effects such as lung fibrosis, scarring, or second malignancies. The theoretical benefits of LDRT, based on the mechanisms discussed earlier, relate to possible effects on hematological cells in the circulation, transit in the heart, or resident cells in the spleen or bone marrow and/or endothelial cells (43,44). If the proper subset of these cells could be eliminated, applying LDRT might be a meaningful exercise. It could be argued that in the midst of a global pandemic with no known intervention (aside from remdesivir and dexamethasone) that has statistically significantly reduced severity of symptoms or mortality to date, the use of LDRT with contemporary radiation techniques inclusive of computed tomography-based planning and modern radiation dosimetry may offer a simple and readily accessible intervention. Although there is always a concern with use of radiation for any clinical disease, the cost of not receiving these treatments may be far worse than that of receiving these treatments, especially when LDRT is reserved for moderately symptomatic (oxygen-dependent) and severely affected (intubated) patients who have a mounted a dysfunctionally exuberant immune response and for whom the risk–benefit ratio is favorable. Mounting evidence suggests that severe COVID infections are associated with elevated neutrophil to lymphocyte ratios, CRP, and IL-6 and reduced lymphocyte and monocyte counts but a relative increase in exhausted (Tim3 and PD1 positive) CD8+ T cells and highly inflammatory CD14 +CD16+ monocytes (45).

The primary concern with LDRT is the potential lack of efficacy as detailed in some reports above. Because lymphocytes are also required to mount an immune response against COVID-19, excessive inactivation of lymphocytes may theoretically impair the ability to mount a counteroffensive to the virus and hence hasten mortality. Undoubtedly, the dose and volume of LDRT would influence the degree of lymphocytic inactivation, but currently there are no validated countermeasures for radiation-induced lymphopenia. Furthermore, there may be a theoretical risk of radiation-induced mutations of the viral genome that can potentially induce selection pressure, leading to unintended and undesirable evolutionary changes during viral replication. Another disadvantage of LDRT use for the current global health pandemic relates to logistical aspects of RT delivery. Transportation of a known COVID-19 patient to radiotherapy departments, along with the process of computed tomography simulation and RT delivery can risk infection of staff and other patients. Moreover, the time required to adequately sterilize RT equipment between patients may limit the ability to treat other patients in a given time frame. Many clinicians and radiation therapists may also not be comfortable with treating intubated patients. For all these reasons, consideration of LDRT for COVID-19 is logistically, mechanistically, and ethically challenging, warranting careful consideration of the pros and cons of invoking such a treatment approach.

Nevertheless, multiple studies across several nations are now underway to assess the impact of LDRT (Table 1). Two of these are available in preprint or print format. A preprint version of the trial from Emory University evaluated 1.5-Gy whole-lung LDRT in 10 hospitalized and oxygen-dependent COVID-19 patients. The median time to clinical recovery was 3 days compared with 12 days for 10 matched control patients, 6 of whom received COVID-directed therapy (*P* = .05) (46). This was also associated with a shorter time to hospital discharge (12 vs 20 days), lower intubation rates (10% vs 40%), and faster radiographic improvement. They also noted faster recovery of serum hematologic, cardiac, hepatic, clotting, and inflammatory markers. Similar findings have now been reported from Iran, where 5 patients older than 60 years of age and requiring supplemental oxygen were treated with 50 cGy of whole-lung radiation, and 4 of them had improvement in oxygen saturation and body temperature within a day, with IL-6 and CRP values also showing a similar trend (45). The ongoing studies predominantly target patients meeting World Health Organization criteria for severe pneumonia before progressing to ventilator dependence and use a range of doses up to 150 cGy. A variety of endpoints are included, with the predominant goal being to reduce pulmonary compromise (oxygen requirement), progression to intubation, and/or duration of intensive care unit stay. Very recently, a planned interim analysis of a single-institution phase I/II trial of a single-fraction, low-dose, whole-lung radiation treatment for hospitalized COVID-19 patients was reported as a preprint abstract (47). Five clinically deteriorating patients requiring supplemental oxygen for radiographically evident pneumonic infiltrates who received radiation noted an improvement in median Glasgow Coma Score from 10 to 14, with 4 exhibiting radiographic improvement and 4 being weaned off oxygen within 4 days. Notably, there were no appreciable toxicities, and 3 patients were weaned off oxygen within 24 hours of radiation. Rapid clinical improvement noted in this study may justify continued evaluation across a range of indications, from early pneumonia requiring oxygen to late stages requiring intubation or intensive care unit stays.

**Table 1.** Overview of ongoing and reported trials of LDRT for COVID-19^a^

Trial details	Patients, No.	Age, y	Requiring O_2_ supplementation?	Whole-lung radiation dose	Outcome metric
RESCUE 1-19 (Emory)	10	≥18	Yes	150 cGy	Safety Clinical recovery
Imam Hossein Hospital (Iran)	5	>60	Yes	50 cGy (+ optional 50 cGy)	SaO_2_ Length of hospital/ICU stay
COLOR-19 (Italy)	30	≥50	Yes	70 cGy	Length of hospital stay Clinical recovery
VENTED (Ohio State University)	24	≥18	Yes (ventilated)	80 cGy	30-d mortality
All India Institute trial	10	≥18	No (but NEWS ≥ 5)	70 cGy	Symptom improvement (NEWS), 30-d ICU admission rate and mortality
Hospital La Milagrosa (Spain)	15	>18	Yes	80 Cgy	Oxygen therapy deescalation SaO_2_

^a^COVID-19 = coronavirus disease-2019; ICU = intensive care unit; LDRT = low-dose radiation therapy; NEWS = National Early Warning Score; SaO_2_ = oxygen saturation.

A convergence of evidence suggests that the proximate cause of deteriorating pulmonary status in COVID-19 patients is an unchecked and ineffective immune response to the viral infection that causes a cytokine storm. Promising preclinical data show that LDRT may attenuate immune activation in other settings and could explain some of the observed benefit in the past with infectious pneumonias. This historical precedent of use of LDRT to treat viral pneumonias, coupled with our current understanding of COVID-19 pathophysiology and LDRT-mediated immune suppression serves as the impetus for consideration of LDRT for COVID-19. In the absence of controlled clinical trials, it remains unclear whether LDRT can reverse the clinical course of patients and meaningfully affect morbidity and/or mortality. Prospective clinical trials, preferably with immune and other normal tissue correlates, appear promising thus far and will hopefully help answer this question in the coming months.

## Funding

None.

## Notes

**Role of the funder: **Not applicable.

**Disclosures:** The authors have no conflicts of interest to disclose.

**Disclaimer:** This commentary is not a statement of policy or the view of the Division of Cancer Treatment and Diagnosis or the National Cancer Institute. It reflects the personal opinions of the authors (ES and MMA) based on prior academic research affiliations.

**Author contributions:** B.P.V. and S.K. conceived of the presented idea. B.P.V., S.L., C.H., V.V., E.S., M.A., and S.K. discussed the structure and format of the manuscript; contributed to writing of the first drafts; and reviewed, commented on, and approved the final manuscript.

## Data Availability

The data underlying this article are available in the article.
